# The tumor suppressive miR-302c-3p inhibits migration and invasion of hepatocellular carcinoma cells by targeting TRAF4: Erratum

**DOI:** 10.7150/jca.76866

**Published:** 2022-08-19

**Authors:** Liu Yang, Yang Guo, Xin Liu, Tongtong Wang, Xiangmin Tong, Kefeng Lei, Jiahui Wang, Dongsheng Huang, Qiuran Xu

**Affiliations:** 1 Key Laboratory of Tumor Molecular Diagnosis and Individualized Medicine of Zhejiang Province, Zhejiang Provincial People's Hospital (People's Hospital of Hangzhou Medical College), Hangzhou, Zhejiang 310014, China; 2 Bengbu Medical College, Bengbu, Anhui 233030, China; 3 Department of Neurosurgery, Zhejiang Provincial People's Hospital (People's Hospital of Hangzhou Medical College), Hangzhou, Zhejiang 310014, China; 4 ZheJiang Chinese Medical University, Hangzhou, Zhejiang 310014, China; 5 Department of Gynecology, Zhejiang Provincial People's Hospital (People's Hospital of Hangzhou Medical College), Hangzhou, Zhejiang 310014, China; 6 School of Basic Medical Sciences, Shandong University, Jinan, Shandong 250000, China

We regret that the original version of our paper unfortunately contained some incorrect representative images. The images of migrated and invaded MHCC97H cells with miR-302c-3p mimics transfection in Figure 3B were mis-pasted. The wrong images were placed in the miR-302c-3p mimics group in Figure 3B when choosing representative images from the countless image data. The correct version of the Figure 3B appears below. The authors confirm that the corrections made in this erratum do not affect the original conclusions. All the authors of the paper have agreed to this correction. The authors apologize for any inconvenience that the errors may have caused.

## Figures and Tables

**Figure 3:**
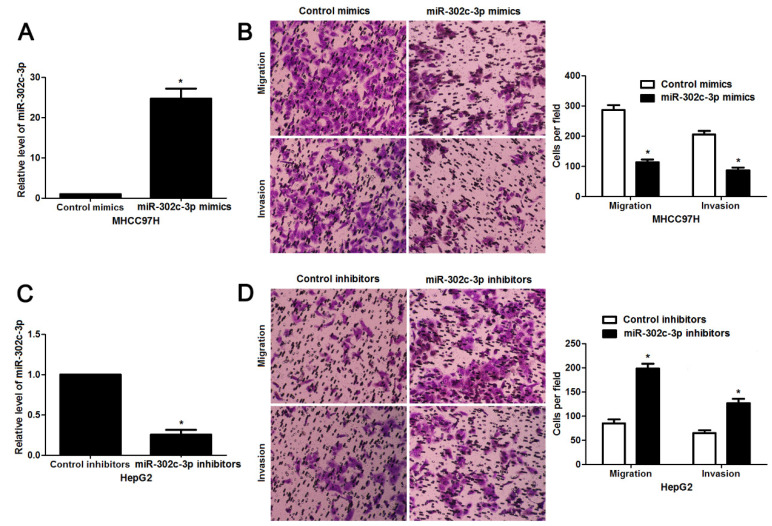
**miR-302c-3p inhibits migration and invasion of HCC cells.** (A) MHCC97H cells that were transfected with miR-302c-3p mimics and control mimics, respectively, were subjected to qRT-PCR for miR-302c-3p expression. n= three repeats with similar results, *P<0.05 by Student's t-test. (B) Transwell assays indicated that miR-302c-3p overexpression suppressed the migration and invasion of MHCC97H cells. n= three repeats with similar results, *P<0.05 by Student's t-test. (C) HepG2 cells that were transfected with miR-302c-3p inhibitors and control inhibitors, respectively, were detected by qRT-PCR for miR-302c-3p expression. n= three repeats with similar results, *P<0.05 by Student's t-test. (D) The migration and invasion capacities of HepG2 cells was enhanced by miR-302c-3p knockdown. n= three repeats with similar results, *P<0.05 by Student's t-test.

